# Microbe‐induced resistance involves priming of direct or indirect defenses according to the stage of herbivory

**DOI:** 10.1111/nph.70652

**Published:** 2025-10-29

**Authors:** Javier Rivero, Iván Fernández, Francisco J. Colina, Axel J. Touw, Alexander Weinhold, Pablo M. Rodríguez‐Blanco, Karen Kester, María J. Pozo, Nicole M. van Dam, Ainhoa Martínez‐Medina

**Affiliations:** ^1^ Department of Soil and Plant Microbiology Estación Experimental del Zaidín – Consejo Superior de Investigaciones Científicas (EEZ‐CSIC) Granada 18008 Spain; ^2^ German Centre for Integrative Biodiversity Research (iDiv) Halle‐Jena‐Leipzig Leipzig 04103 Germany; ^3^ Laboratory of Molecular Agroecology Estación Experimental del Zaidín – Consejo Superior de Investigaciones Científicas (EEZ‐CSIC) Granada 18008 Spain; ^4^ Institute of Biodiversity, Ecology and Evolution (IBEE), Friedrich Schiller University Jena Jena 07743 Germany; ^5^ Leibniz Institute of Vegetable and Ornamental Crops (IGZ) Großbeeren 14979 Germany; ^6^ Plant‐Microorganism Interactions Institute of Natural Resources and Agrobiology of Salamanca – Consejo Superior de Investigaciones Científicas (IRNASA‐CSIC) Salamanca 37008 Spain; ^7^ Department of Biology Virginia Commonwealth University Richmond Virginia 23284 USA

**Keywords:** arbuscular mycorrhiza, microbe‐induced resistance, multitrophic interactions, plant–microbe–insect interactions, priming, tomato, *Trichoderma*

## Abstract

- Microbe‐induced resistance (MIR) encompasses a broad range of plant responses that mediate both direct and indirect defenses against herbivores. However, previous studies have addressed MIR in relation to either direct or indirect defenses in isolation, overlooking potential conflicts or synergies that may arise between MIR‐elicited defense strategies.
- We hypothesize that in multitrophic contexts, MIR‐driven elicitation of direct and indirect defenses interacts, thereby shaping overall MIR phenotypes. To test this, we used two MIR‐eliciting fungi: an arbuscular mycorrhizal and *Trichoderma* species; and the tri‐trophic system including tomato plants, the herbivore *Manduca sexta*, and the parasitoid *Cotesia congregata*. We conducted a series of MIR bioassays, including performance and behavioral assays, and explored the main mechanisms underlying MIR phenotypes.
- We found that MIR involves a dynamic elicitation of direct and indirect defense‐related traits that plastically adjust to herbivory time. Upon short‐term herbivory, MIR primed jasmonate‐regulated defenses, thereby enhancing the mortality of early‐instar larvae. After a longer period of herbivory, MIR, especially triggered by *Trichoderma*, boosted green leaf volatile emissions, enhancing parasitoid attraction. Parasitoid performance was improved by MIR.
- Our study revealed that MIR enhances resistance by priming both direct and indirect defenses at different herbivory stages. This fosters complementary and synergistic responses, enhancing MIR phenotypes.

## Resumen

- La resistencia inducida en plantas por microorganismos (MIR, por sus siglas en inglés) frente a insectos herbívoros implica la activación de respuestas defensivas tanto directas como indirectas. En general, los estudios previos han analizado la contribución de las defensas directas o indirectas al funcionamiento de MIR de manera aislada, sin considerar los posibles conflictos o sinergias que podrían surgir entre ambas estrategias.
- Nuestra hipótesis es que, en contextos multitróficos, las defensas directas e indirectas activadas por MIR pueden interactuar, determinando el fenotipo global de resistencia inducida. Para poner a prueba esta hipótesis, utilizamos dos hongos beneficiosos del suelo inductores de MIR: un hongo formador de micorrizas arbusculares y un hongo del género *Trichoderma*, junto con un sistema tritrófico compuesto por plantas de tomate, el herbívoro *Manduca sexta* y el parasitoide *Cotesia congregata*. Realizamos una serie de bioensayos en invernadero que incluyeron ensayos de desempeño y comportamiento de herbívoros y enemigos naturales, y analizamos los principales mecanismos de defensa de la planta que subyacen a los fenotipos de MIR.
- Nuestros resultados muestran que el funcionamiento de MIR implica una inducción dinámica de defensas directas e indirectas, que se ajusta de manera plástica según la duración de la herbivoría. Tras un periodo corto de herbivoría, MIR potenció las defensas de la planta reguladas por jasmonatos, aumentando la mortalidad de las larvas en estadios tempranos. Después de un periodo prolongado de herbivoría, MIR, especialmente la inducida por *Trichoderma*, incrementó la emisión de volátiles de hojas verdes, favoreciendo la atracción de los parasitoides. Además, MIR mejoró el desempeño de estos enemigos naturales.
- En conjunto, nuestro estudio revela que MIR refuerza la resistencia de las plantas al potenciar de manera dinámica tanto las defensas directas como las indirectas en distintas etapas de la herbivoría, promoviendo respuestas complementarias y sinérgicas que fortalecen los fenotipos de resistencia.

## Introduction

Plants possess a diverse suite of defensive traits, which have captivated biologists for centuries. Among these strategies, induced defenses represent a form of phenotypic plasticity that enables plants to adjust their morphological and metabolic phenotypes to environmental variations (Kessler & Mueller, 2024). Following an attack, plants respond to herbivory through changes in secondary metabolism, leading to an increased production of repellent, toxic, and antinutritive compounds that reduce herbivore survival and reproductive success. These are referred to as direct defenses (Erb & Kliebenstein, 2020). Simultaneously, herbivory also induces the production and release of volatile organic compounds (VOCs), which can serve as cues to natural enemies of herbivores (the third trophic level), thereby facilitating prey‐searching behavior and mediating the so‐called indirect defenses (Pearse *et al*., 2020). In multitrophic communities, both direct and indirect inducible defenses co‐occur and play major roles in shaping plant defense phenotypes. Ultimately, this influences the outcome of plant–herbivore interactions (Agrawal & Fishbein, 2006; Agrawal, 2011). Still, the temporal dynamics and interplay between direct and indirect defense strategies are highly complex, encompassing both synergistic interactions and antagonistic trade‐offs (Rasmann & Agrawal, 2009; Rasmann *et al*., 2011; Fatouros *et al*., 2014; Edwards *et al*., 2023; Graham *et al*., 2023).

In addition to relying on an intrinsic immune system, plants engage with a variety of mutualistic microbes that significantly contribute to their defense phenotypes. Plant–microbe mutualistic associations are prevalent in nearly all terrestrial ecosystems and include those with rhizobacteria and rhizofungi (Pieterse *et al*., 2014; Pozo *et al*., 2021). Root‐associated mutualistic microbes can modulate host immune responses, resulting in enhanced protection against pathogens and pests, a phenomenon known as Microbe‐Induced Plant Resistance (MIR, also referred to as Induced Systemic Resistance, ISR) (Pieterse *et al*., 2014). MIR often functions by ‘sensitizing’ the plant's immune system, leading to boosted activation of defenses upon pathogen or herbivore attack. This mechanism, referred to as defense priming, provides the plant with a cost‐effective form of protection (Martínez‐Medina *et al*., 2016). For example, root colonization by arbuscular mycorrhizal (AM) or *Trichoderma* fungi primes leaves for a boosted accumulation of antiherbivore secondary metabolites, thereby reducing herbivore performance (Contreras‐Cornejo *et al*., 2018; Alınç *et al*., 2021; Papantoniou *et al*., 2021; Rivero *et al*., 2021; Lidoy *et al*., 2025). In addition, several studies evidenced that AM and *Trichoderma* fungi can impact VOC release in response to herbivory, promoting plant interactions with the third trophic level and thus indirect defenses (Fontana *et al*., 2009; Schausberger *et al*., 2012; Battaglia *et al*., 2013; Babikova *et al*., 2014; Coppola *et al*., 2017; Papantoniou *et al*., 2022). By enhancing the plant's defensive capabilities, MIR can have significant effects on the outcomes of plant–herbivore interactions across multiple trophic levels and, by extension, on population and community dynamics (Martínez‐Medina *et al*., 2024). However, despite the critical role of MIR in plant–herbivore dynamics, the mechanisms underlying MIR regulation and functioning, particularly in multitrophic contexts, remain largely unknown.

While it is well established that MIR significantly influences plant immune responses to herbivores, research has mainly been restricted to assessing the impact of MIR on either direct or indirect defense lines in isolation. However, MIR enhancement of direct defenses may significantly influence the expression of indirect defenses, and vice versa. These interactions may potentially lead to complex, yet unexplored, consequences for plant–insect interactions. For example, given that plant resources are limited, plants may face allocation trade‐offs when generating different lines of induced defense responses (Kessler & Heil, 2011; Agrawal & Hastings, 2019). Consequently, MIR enhancement of direct defenses might constrain the resources available for the expression of indirect defenses, potentially resulting in MIR‐associated allocation costs. Moreover, MIR‐boosted accumulation of toxic chemicals in leaves might negatively impact the development of natural enemies feeding on herbivores. This may be especially true when these compounds are transferred to the third trophic level through herbivores that accumulate or sequester them, potentially leading to MIR‐associated ecological costs (Kessler & Heil, 2011; Martínez‐Medina *et al*., 2024). Alternatively, MIR might promote complementary and synergistic interactions between direct and indirect inducible defenses, leading to reduced herbivore pressure. For instance, MIR‐boosted direct defenses might support indirect defenses by weakening the immune or behavioral defense responses of herbivores to parasitoid or predator attacks (Martínez‐Medina *et al*., 2024). Remarkably, explicit tests of whether the elicitation of direct and indirect defenses integrates during MIR are lacking. As a result, it remains unclear whether and to what extent this integration may influence MIR phenotypes and, eventually, plant–insect multitrophic interactions. This knowledge gap may stem from the logistical challenges associated with conducting such studies, which require integrating molecular, physiological, performance, and behavioral analyses across complex biological systems involving microbes, plants, herbivores, and natural enemies.

To better understand how MIR phenotypes function in multitrophic contexts, we explicitly investigated how and to what extent MIR expression simultaneously affects direct and indirect plant defenses. We hypothesize that in multitrophic systems, MIR elicitation of direct and indirect defenses operates simultaneously and interacts with one another. Our aim was to assess whether such potential interactions result in antagonistic, neutral, or synergistic effects between both strategies and how this influences the overall functioning of MIR. We used two different well‐characterized and functionally distinct MIR‐eliciting fungi: *Trichoderma harzianum* (a root endophyte) and *Rhizophagus irregularis* (an AM fungus). Additionally, we used a tri‐trophic model system consisting of tomato plants (*Solanum lycopersicum*, first trophic level), the specialist herbivore *Manduca sexta* (second trophic level), and the specialist endoparasitic wasp *Cotesia congregata* (third trophic level).

We found that MIR expression involves priming of both direct and indirect defenses, primarily by enhancing jasmonate production via the allene oxide synthase (AOS) branch of the oxylipin (13‐LOX) pathway and by increasing green leaf volatile emissions via hydroperoxide lyase (HPL) of the 13‐LOX pathway. Strikingly, MIR phenotypes exhibited plastic adjustments at the molecular and physiological levels according to the duration of herbivory. Shortly after the onset of herbivory, MIR was specifically associated with priming the AOS branch. This boosted jasmonate production and its downstream defenses, thereby promoting mortality of early instar larvae. After prolonged herbivory, MIR was mostly associated with priming the HPL branch. This enhanced the production of green leaf volatiles, thereby enhancing parasitoid attraction. We propose that this dynamic adjustment of MIR fosters complementarity between MIR elicitation of direct and indirect defense strategies. Notably, parasitoid performance was enhanced by MIR, also highlighting a synergistic interaction among MIR elicitation of both defense strategies. We argue that the dynamic adjustment of MIR during herbivory represents a new emergent property of MIR that promotes complementarities and synergies between MIR‐elicited direct and indirect defenses, thereby enhancing MIR phenotypes.

## Materials and Methods

### Plant, fungal, and insect material

We used the tomato (*Solanum lycopersicum* L.) cultivar Moneymaker. The inoculum of the arbuscular mycorrhizal fungus *Rhizophagus irregularis* (Błaszk., Wubet, Renker & Buscot; C. Walker & A. Schußler) was purchased from INOQ GmbH, Germany (https://inoq.de), containing 220 mycorrhizal units ml^−1^ of sand. The root endophytic fungus *Trichoderma harzianum* (Rifai) was maintained on potato dextrose agar at 4°C. The *T. harzianum* inoculum was prepared according to Martínez‐Medina *et al*. (2009). Eggs of *Manduca sexta* (L.; Lepidoptera: Sphingidae) were obtained from the laboratory colony at the Max Planck Institute for Chemical Ecology (MPI‐CE), Jena, Germany. This colony is maintained under laboratory conditions on an artificial diet according to Grosse‐Wilde *et al*. (2011). We kept the eggs and larvae in a plexiglass cage at 27°C, with a 16 h : 8 h, light : dark cycle, and at 50% relative humidity. Cocoons of *C. congregata* (Say; Hymenoptera: Braconidae) wasps were obtained from a laboratory colony maintained at Virginia Commonwealth University, as described by Kester & Barbosa (1991). We maintained the cocoons in a plexiglass cage at 22° ± 2°C, with a 12 h : 12 h, light : dark cycle, and 50% relative humidity. After emergence, adult wasps were maintained at the same conditions and fed with a honey‐sterile water solution 1 : 5 (v/v) on cotton pads.

### Plant growth conditions and fungal inoculation

Tomato seeds were surface‐sterilized in 4% NaClO_4_, rinsed thoroughly with sterile water, and germinated for 1 wk in vermiculite at 25°C. Tomato seedlings were then transferred to 400‐ml pots containing a sterile sand : vermiculite (1 : 1, v/v) mixture. Next, they were assigned to one of the three treatment groups: noninoculated plants, plants inoculated with *R. irregularis*, and plants inoculated with *T. harzianum*. Inoculation with *R. irregularis* was performed by mixing the inoculum with the substrate at 10% (v/v) before transplanting (Papantoniou *et al*., 2021). Inoculation with *T. harzianum* was achieved by mixing the inoculum with the substrate to a final density of 1 × 10^6^ conidia g^−1^ before transplanting (Papantoniou *et al*., 2021). The plants were then placed in a completely randomized design in a glasshouse compartment with a 25 ± 3°C, 16 h : 8 h, light : dark cycle, and 70% relative humidity. The plants were watered three times a week: 2 d using tap water and 1 d using 50% Hoagland nutrient solution (Hoagland & Arnon, 1950), which contained 25% of the standard phosphorus. Four weeks after transplanting, the plants were used for experiments.

### Assessment of herbivore performance

Four weeks after transplanting, two *M. sexta* neonates were placed on the third fully expanded leaf (counted from the top) and allowed to feed *ad libitum* on the entire plant. Individual plants were covered with mesh bags. The plants had been inoculated with *R. irregularis* or *T. harzianum*, or remained uninoculated as described above, with 25 biological replicates (plants) per treatment group. The weight and survival of the *M. sexta* larvae were assessed periodically over 16 d.

### Assessment of jasmonate accumulation, gene expression, and volatile emission

Four weeks after transplanting, plants were infested with *M. sexta* larvae. The plants had been inoculated with *R. irregularis* or *T. harzianum*, or remained uninoculated as described above, with *n* = 6 plants per treatment group and time point. For short‐term herbivory treatments (1‐d herbivory, hereafter short‐term herbivory), two first‐instar *M. sexta* larvae were placed on the apical leaflet of the third fully expanded leaf (counted from the top) and confined using a clip cage. For long‐term herbivory (10‐d herbivory, hereafter long‐term herbivory), one first‐instar *M. sexta* larva was placed in the clip cage and moved through the plant every two or three days to avoid the consumption of the entire leaf. To ensure a similar level of damage across treatments throughout the entire bioassay, dead *M. sexta* larvae were immediately replaced by larvae of the same age that had been feeding on extra plants from the same treatment group. Empty clip cages were placed on the leaves of uninfested plants. At the first and 10th day after *M. sexta* infestation, leaf volatiles were sampled, and leaf material was collected and stored at −80°C for phytohormone and gene expression analyses. For short‐term herbivory, volatile sampling and leaf material collection were performed on the same damaged apical leaflets, whereas for long‐term herbivory, volatile sampling and leaf material collection were performed on apical leaflets that had been damaged within the last 24 h of the 10‐d feeding period. For undamaged plants, volatile sampling and leaf material collection were performed similarly. Root material and substrate attached to the root system were collected and reserved for microbiological analyses. We selected five replicates per treatment for subsequent analyses of jasmonate accumulation, gene expression, and volatile emission.

### Fungal quantification

We confirmed that *R. irregularis* had efficiently colonized tomato roots in all the bioassays performed by washing the roots in KOH (10%) and subsequently staining fungal structures with 5% ink in 2% acetic acid (Vierheilig *et al*., 2005). The extent of mycorrhizal colonization was calculated according to the gridline intersection method (Giovannetti & Mosse, 1980) using a Leica S8APO stereomicroscope. The extent of mycorrhizal colonization in *R. irregularis*‐inoculated plants ranged from 30% to 40% (percentage of total root length colonized by *R. irregularis*). We confirmed that *T. harzianum* had efficiently colonized the potting substrate in all the performed bioassays by using the plate count technique and potato dextrose agar amended with 50 mg l^−1^ rose bengal and 100 mg l^−1^ streptomycin sulfate according to Papantoniou *et al*. (2021). The number of *T. harzianum* CFU in the potting media of *T. harzianum*‐inoculated plants was similar to the initial inoculation values (i.e. 1 × 10^6^ conidia g^−1^).

### Real‐time quantitative reverse transcription PCR

Total RNA was extracted from fresh leaves of five random independent replicates per treatment. RNA isolation, first‐strand cDNA synthesis, quantitative PCR, and data processing were conducted as described in Supporting Information Methods S1, using the gene‐specific primers listed in Table S1.

### Determination of jasmonate concentrations

We extracted plant hormones from fresh leaves of five random independent replicates per treatment. We used the same five replicates as for the transcriptional analyses. Hormone extraction, analysis, and data processing were performed as described in Methods S2.

### Volatile collection, gas chromatography, and volatile data processing

Leaf volatiles were sampled in the glasshouse at 25 ± 3°C and 70% relative humidity, between 10:00 h and 14:00 h, for each time point. VOC analysis was performed using the same five replicates as for the transcriptional and metabolomic analyses. Volatile collection, analysis, and data processing were performed as described in Methods S3.

### Assessment of parasitoid behavior

We used 2‐ to 4‐d‐old, mated but naive female *C. congregata* wasps. Wasps were sexed based on morphological parameters under a Leica S8APO stereomicroscope. We performed dual‐choice tests using a closed‐system Y‐tube olfactometer, which was illuminated from above as described in Methods S4. Plants were treated as described above in the bioassay for ‘Assessment of jasmonate accumulation, gene expression, and volatile emission’. To confirm that *C. congregata* can use volatiles from *M. sexta*‐infested tomato plants as host‐location cues in our experimental setup, we first performed two dual‐choice experiments: uninfested plants vs plants infested with *M. sexta* upon short‐term herbivory (1 d of continuous feeding), and uninfested plants vs plants infested with *M. sexta* upon long‐term herbivory (10 d of continuous feeding). To test the impact of *R. irregularis* and *T. harzianum* on the attraction of the wasps, we included the following dual‐choice experiments during *M. sexta* short‐term and long‐term herbivory: noninoculated plants vs *R. irregularis*‐inoculated plants; and noninoculated plants vs *T. harzianum*‐inoculated plants. To assess whether *C. congregata* can also use volatiles from uninfested *R. irregularis*‐inoculated or *T. harzianum*‐inoculated plants as host‐location cues, the following experiments were further conducted with uninfested plants: noninoculated plants vs *R. irregularis*‐inoculated plants, and noninoculated plants vs *T. harzianum*‐inoculated plants. For the entire study, we tested *c*. 600 female wasps.

### Assessment of parasitoid performance

We assessed parasitoid development on *M. sexta* caterpillars reared on plants that had been inoculated with *R. irregularis*, *T. harzianum*, or not inoculated. Parasitization of *M. sexta* larvae by *C. congregata* wasps was performed as detailed in Methods S5. A total of 60 *M. sexta* larvae were parasitized, and their development was monitored on noninoculated plants (*n* = 20), *R. irregularis*‐inoculated plants (*n* = 20), and *T. harzianum*‐inoculated plants (*n* = 20).

### Statistical analysis

Datasets were analyzed using the R software v.3.6.1 (R Core Team, 2021) and the Metaboanalyst web server v.4.0 (Xia & Wishart, 2016) as described in Methods S6.

## Results

### The expression of MIR results in increased mortality of early‐instar *M. sexta* larvae

**Fig. 1:**
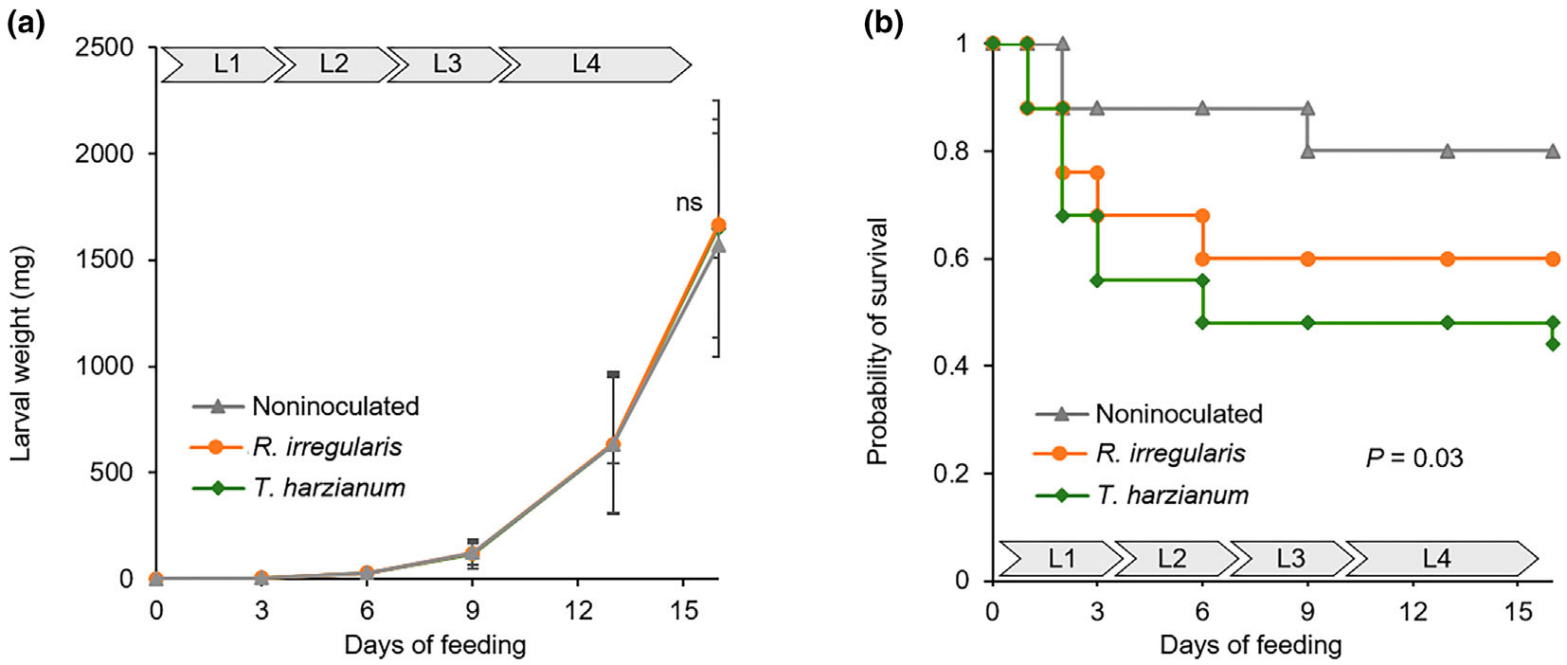
Impact of tomato root colonization by *Rhizophagus irregularis* or *Trichoderma harzianum* on the weight and probability of survival of *Manduca sexta* larvae. In (a), the weight of *M. sexta* larvae, and in (b), the probability of survival were assessed periodically for individuals feeding from leaves of tomato plants that had been inoculated with *R. irregularis* or *T. harzianum*, or noninoculated. Arrow bars in each panel represent the larval developmental stage, according to the feeding time, from instar L1 to instar L4. In (a), the data points represent the average of 25 replicates (plants) ± SD. In (b), Kaplan–Meier survival curves indicate the probability of survival (*n* = 50 per treatment group). Significant differences between treatments were calculated by the log‐rank test. ns, nonsignificant differences.

We conducted a bioassay to determine whether *R. irregularis* or *T. harzianum* root colonization affects the performance of *M. sexta* larvae. Larvae fed on noninoculated plants exhibited exponential growth from day 9 onward, coinciding with reaching the fourth larval instar (L4, Fig. 1a). Root colonization by *R. irregularis* or *T. harzianum* did not significantly affect larval weight gain (Fig. 1a). However, root colonization led to a significant decrease in the survival rates of *M. sexta* larvae, compared with those in noninoculated plants (Fig. 1b). This microbe‐induced decrease in survival was the strongest over the first 6 d of herbivory, corresponding with larval instars L1–L3 (Cox regression performed on data from the first 6 d for *R. irregularis* treatment: exp(coef) = 3.91; *P* = 0.038; for *T. harzianum* treatment exp(coef) = 5.49; *P* = 0.007). From day 6 onward, the survival rates of the then L4–L5 larvae did not significantly decrease anymore (Cox regression performed on data from day 6 onward for *R. irregularis* treatment: exp(coef) = 1.37; *P* = 0.751; for *T. harzianum* treatment exp(coef) = 2.5; *P* = 0.306). These findings indicate that MIR, triggered by *R. irregularis* or *T. harzianum*, leads to increased mortality of *M. sexta* larvae specifically during early‐larval instars (i.e. from L1 to L3).

### The expression of MIR results in increased attraction of the parasitoid *C. congregata* specifically upon long‐term herbivory

**Fig. 2:**
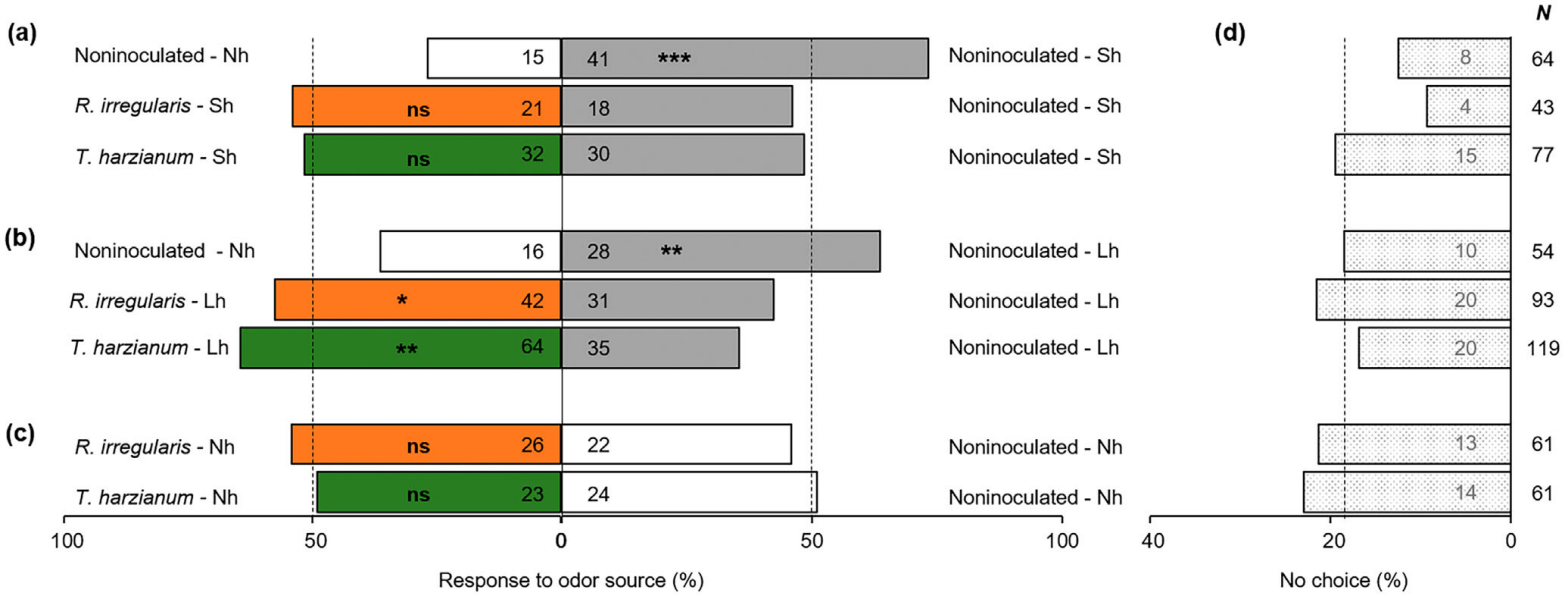
Behavioral responses in a Y‐tube olfactometer of *Cotesia congregata* female parasitoids to volatiles from differently treated tomato plants. Plants were root‐inoculated with *Rhizophagus irregularis* or *Trichoderma harzianum*, or noninoculated. Plants were challenged with *Manduca sexta* larvae for 1 d (short‐term herbivory, Sh), or for 10 d (long‐term herbivory, Lh), or kept without herbivores (no herbivory, Nh). Data represent the percentage of parasitoids that chose either of the two odor sources, as determined with different sets of plants. (a) Results from the dual‐choice tests after short‐term herbivory; (b) idem after long‐term herbivory; (c) dual‐choice test with plants without herbivores. The number of parasitoid females that chose either of the two odor sources is given for each test. (d) The number (*N*) of tested parasitoids in each test, and the number and percentage of parasitoids that did not make a choice. Asterisks indicate a significant difference within a choice test: *, *P* < 0.05; **, *P* < 0.01; ***, *P* < 0.001. ns, nonsignificant differences (binomial test).

We conducted dual‐choice olfactometer assays to assess whether root colonization by *R. irregularis* or *T. harzianum* affects the attraction of the parasitoid *C. congregata*. We considered two different time points following the onset of herbivory: 1 d after herbivore feeding (short‐term herbivory) and 10 d after herbivore feeding (long‐term herbivory). In a setup with noninoculated plants only, we first confirmed that *C. congregata* wasps prefer volatiles of *M. sexta*‐damaged plants both after short‐term herbivory (Sh) and long‐term herbivory (Lh), compared with those from undamaged plants (Nh) (Fig. 2a,b). In bioassays with only undamaged plants (Nh), wasps did not discriminate between volatiles emitted from noninoculated plants and those emitted from *R. irregularis*‐colonized or *T. harzianum*‐colonized plants (Fig. 2c).

A comparison of treatments involving short‐term herbivory (Sh) showed that root colonization by *R. irregularis* or *T. harzianum* did not significantly affect the attraction of parasitoids. Indeed, parasitoids were equally attracted to noninoculated and *R*. *irregularis*‐*colonized* or *T. harzianum*‐colonized plants (Fig. 2a). By contrast, we observed a higher proportion of parasitoid wasps choosing *R. irregularis*‐colonized plants and *T. harzianum*‐colonized plants over noninoculated plants when these plants were subjected to long‐term herbivory (Lh) (Fig. 2b). The effect of *T. harzianum* colonization on the attraction of parasitoid wasps was stronger than that of *R. irregularis*. Our findings indicate that MIR, triggered by *R. irregularis* or *T. harzianum*, increases the attraction of *C. congregata* wasps toward volatiles emitted by plants subjected specifically to long‐term herbivory, while no MIR effect was detected upon short‐term herbivory.

### The expression of MIR results in enhanced performance of the parasitoid *C. congregata*

**Fig. 3:**
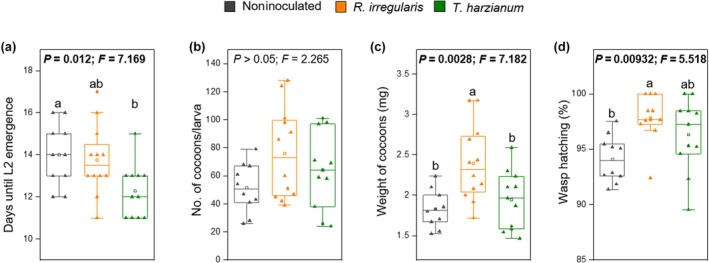
Performance of *Cotesia congregata* when developing *in Manduca sexta* larvae feeding on noninoculated plants or in plants root‐colonized by *Rhizophagus irregularis* or *Trichoderma harzianum*. The effect of root colonization was assessed on (a) time (days) from *C. congregata* oviposition to the emergence of *C. congregata* L2 larvae from *M. sexta* individuals; (b) number of *C. congregata* cocoons within *M. sexta* individuals; (c) fresh weight of *C. congregata* cocoons; and (d) percentage of *C. congregata* wasps hatching from cocoons. Box plots represent the interquartile range (IQR), the bisecting line represents the median, the whiskers represent 1.5 times the IQR, and the dots represent data points (*n* = 10–12 individual *M. sexta* larvae). In (a), (c), and (d), different letters indicate differences between treatments (one‐way ANOVA, Tukey's test; *P* < 0.05). *P* and *F* values at the top of the graph panels indicate the result of the one‐way ANOVA, with treatment as explanatory factor. Lines in bold: *P* < 0.05.

To assess whether root colonization by the mutualistic fungi affects *C. congregata* performance, we evaluated the development of *C. congregata* in third‐instar *M. sexta* larvae reared on inoculated or noninoculated plants. We found that 40–50% of parasitized *M. sexta* individuals did not survive *C. congregata* parasitism, regardless of fungal inoculation. When assessing the performance of the parasitoids developing in the surviving *M. sexta* individuals, we found that *T. harzianum* root colonization reduced the time from parasitoid oviposition to the emergence of L2 parasitoid larvae compared with that in noninoculated plants (Fig. 3a). However, *T. harzianum* inoculation did not affect the number or weight of the cocoons nor the percentage of hatching wasps, compared with noninoculated plants (Fig. 3b–d). By contrast, *R. irregularis* root colonization did not significantly affect parasitoid development (Fig. 3a), but increased the weight of the cocoons and the emergence of wasps (Fig. 3c,d). Overall, these results support that the expression of MIR triggered by *R. irregularis* or *T. harzianum* improves *C. congregata* performance.

### Upon short‐term herbivory, MIR expression is associated with priming of jasmonate‐related defenses

**Fig. 4:**
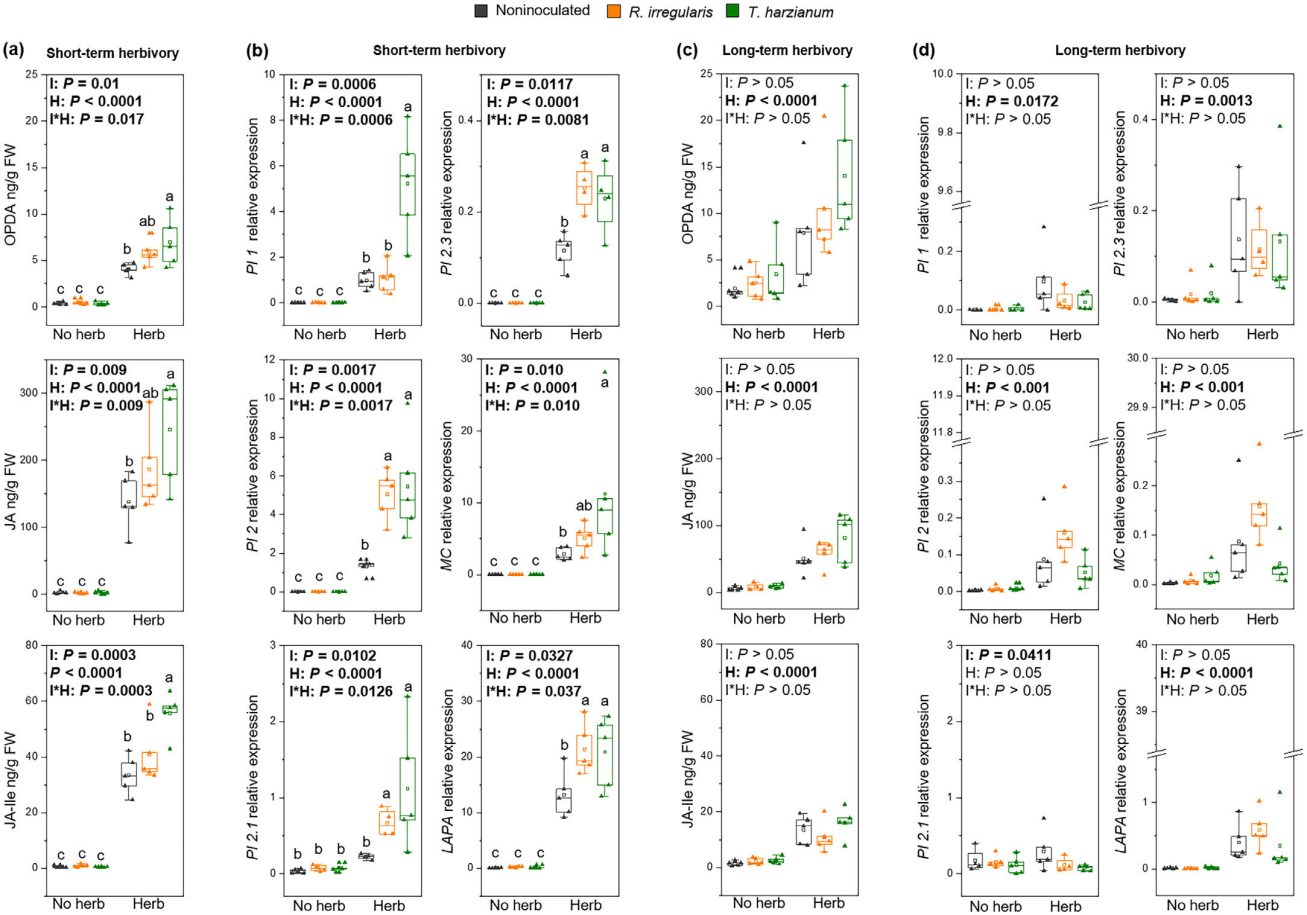
Effect of tomato root colonization by *Rhizophagus irregularis* or *Trichoderma harzianum* on jasmonate‐regulated defenses upon *Manduca sexta* short‐ and long‐term herbivory. In (a, c) levels of OPDA (oxophytodienoic acid), JA (jasmonic acid), and JA‐Ile (jasmonoyl–isoleucine); and in (b, d) expression levels of the JA‐regulated genes *Pi 1*, *Pi 2*, *Pi 2.1*, and *Pi 2.3* (*Protease inhibitors 1*, *2*, *2.1*, and *2.3*), *MC* (*Multicistatin*), and *LAPA* (*Leucine aminopeptidase*). Metabolite accumulation and gene expression were analyzed in leaves of plants subjected to short‐term herbivory (1 d, a, b) or long‐term herbivory (10 d, c, d). In (b, d), the results are normalized to *SlEF* gene expression levels. In each graph, ‘Herb’ stands for plants with herbivores, and ‘No herb’ stands for plants without herbivores. Plants had been inoculated with *R. irregularis* or *T. harzianum*, or noninoculated. Box plots represent the interquartile range (IQR), the bisecting line represents the median, the whiskers represent 1.5 times the IQR, and the dots represent data points from five individual plants (*n* = 5). Different letters in (a) and (b) panels indicate differences between treatments (Tukey's test after two‐way ANOVA; *P* < 0.05). No significant differences were found in (c) and (d) panels. Left upper corner of graph panels: results of two‐way ANOVA. Factors I, inoculation; H, herbivory; and I*H, interaction between inoculation and herbivory. Lines in bold: *P* < 0.05.

Next, we conducted a bioassay to explore whether modulation of the allene oxide synthase (AOS) branch of the 13‐LOX pathway was associated with the observed MIR phenotype (Farmer & Goossens, 2019). We analyzed the levels of the jasmonates: OPDA (oxophytodienoic acid), JA (jasmonic acid), and JA‐Ile (jasmonoyl–isoleucine) in tomato leaves challenged with *M. sexta* larvae, following short‐term (1 d) and long‐term (10 d) continuous herbivory. Additionally, we assessed the expression of defense‐related, jasmonate‐regulated genes encoding the proteinase inhibitors PI1, PI2, PI2.1, and PI2.3; a multicystatin (MC); and a leucine aminopeptidase (LAPA). In the absence of *M. sexta* herbivory, the mutualistic fungi did not affect the accumulation of jasmonates nor the expression level of the analyzed genes (Fig. 4, no herb treatments). Moreover, root colonization by the mutualists did not significantly alter shoot biomass or the number of flowers (Fig. S1). This indicates that root colonization by *R. irregularis* or *T. harzianum* is not associated with the direct activation of the AOS branch of the 13‐LOX pathway nor leads to potential fitness costs. Notably, upon herbivory, root colonization by the mutualistic fungi overall affected jasmonate response depending on the duration of the herbivory (three‐way ANOVA: *P* ≤ 0.05 for JA, JA‐Ile, *PI1*, *PI2*, *PI2.1*, *MC*, and *LAPA*; *P* > 0.05 for OPDA and *PI2.3*; Table S2).

Short‐term herbivory by *M. sexta* triggered the accumulation of jasmonates (Fig. 4a) and the expression of jasmonate‐regulated genes (Fig. 4b). Remarkably, OPDA, JA, and JA‐Ile accumulated to higher levels in leaves of *T. harzianum*‐inoculated plants than of noninoculated plants (Fig. 4a). Accordingly, the jasmonate‐regulated genes *PI1*, *PI2*, *PI2.1*, *PI2.3*, *MC*, and *LAPA* were upregulated more strongly in leaves of *T. harzianum*‐inoculated plants than in noninoculated plants (Fig. 4b). In the case of *R. irregularis*‐inoculated plants, OPDA and JA concentrations tended to be higher after short‐term herbivory, although this effect was not statistically significant, compared to noninoculated plants (Fig. 4a). Moreover, four out of the six jasmonate‐regulated genes were upregulated in *R. irregularis*‐colonized plants in response to herbivory: *PI2*, *PI 2.1*, *PI 2.3*, and *LAPA* (Fig. 4b).

Upon long‐term herbivory, *M. sexta* feeding triggered the accumulation of jasmonates (Fig. 4c) and the expression of the jasmonate‐regulated genes, with the exception of *PI2.1* (Fig. 4d). Upon long‐term herbivory, root colonization by the mutualistic fungi did not significantly affect the accumulation of OPDA, JA, and JA‐Ile anymore (Fig. 4d). Moreover, root colonization did not affect the expression of the jasmonate‐responsive genes upon long‐term *M. sexta* herbivory (Fig. 4d). Notably, herbivory damage level was similar across the different treatments, as shown by similar removed leaf area upon 24 h of herbivory and similar removed biomass upon 10 d of herbivory (Fig. S2). Altogether, our results show that the expression of MIR, triggered by *R. irregularis* and *T. harzianum*, is associated with priming of the AOS branch of the 13‐LOX pathway specifically upon short‐term herbivory, while this effect was not observed upon long‐term herbivory.

### Upon long‐term herbivory, MIR expression is associated with priming of green leaf volatile biosynthesis and emission

We next explored whether root colonization by *R. irregularis* or *T. harzianum* affects the blend of volatile organic compounds (VOCs) released by plants subjected to short‐term (1 d) and long‐term (10 d) continuous herbivory. Across all treatment groups, we detected a total of 118 VOCs released by plants subjected to short‐term herbivory (Table S3) and 71 VOCs during long‐term herbivory (Table S4). Principal component analysis (PCA) revealed a separation between the herbivore‐challenged and nonherbivore‐challenged treatment groups, both at the first and 10^th^ day following herbivory (Fig. S3). We first performed one‐way ANOVA on VOC datasets obtained after long‐term or short‐term herbivory, with plant treatment (including herbivory and microbial inoculation treatments) as the explanatory factor. Upon short‐term herbivory, we found a total of 46 VOCs with significant differences among treatment groups (ANOVA, FDR‐corrected *P* < 0.05; Table S5). Among the top 10 compounds showing significant differences, we found 6 GLVs: (*Z*)‐3‐hexenyl propionate, (*Z*)‐3‐hexenyl butanoate, (*Z*)‐3‐hexenyl acetate, (*E*)‐2‐hexenal, and 2 unknown GLVs with retention times (rt) of 13.14 min and 17.69 min. Additionally, (*2Z*)‐2‐pentenyl butyrate, *cis*‐jasmone, indole, and the monoterpene *E*‐cosmene were among the top 10 differentially regulated compounds. These compounds were enhanced following short‐term herbivory regardless of microbial inoculation, except for *E*‐cosmene, which was reduced. Upon long‐term herbivory, we found only 7 VOCs showing significant differences (ANOVA, FDR‐corrected *P* < 0.05) among treatments, including the 3 GLVs: (*Z*)‐3 hexenyl butanoate, (*Z*)‐3 hexenyl propionate, and (*Z*)‐3‐hexenyl acetate (Table S6). We also found indole, (*2Z*)‐2‐pentenyl butyrate, and the sesquiterpenes dendrolasin and γ‐muurolene to be significantly different among treatments in plants subjected to long‐term herbivory. These compounds were significantly enhanced following short‐term herbivory regardless of microbial inoculation, except for γ‐muurolene, which was reduced.

**Fig. 5:**
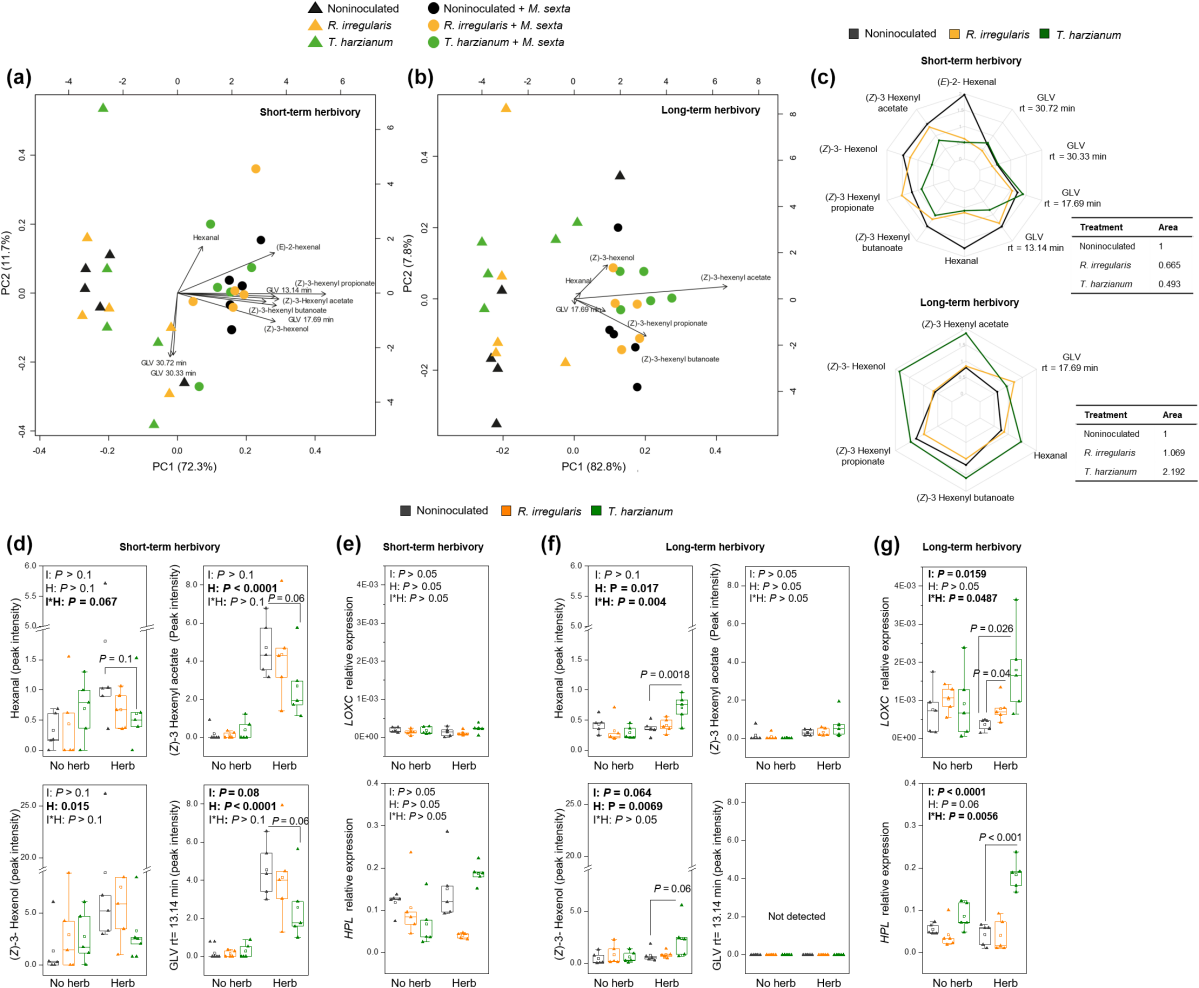
Impact of root colonization by *Rhizophagus irregularis* or *Trichoderma harzianum* on the emission and biosynthesis of green leaf volatiles by tomato plants in response to *Manduca sexta* short‐ and long‐term herbivory. (a, b) Principal component analysis (PCA, *n* = 5) biplot describing the GLVs emitted by leaves of plants without herbivores (triangles), and by plants subjected to short‐term herbivory (1 d, a) or long‐term herbivory (10 d, b) (circles). Plants had been inoculated with *R. irregularis* or *T. harzianum*, or noninoculated. The first two components for each PCA are represented, and the percentage of variation explained is given in parentheses at each axis. The arrows point to the GLVs that contributed to the separation of the samples based on our targeted analysis. (c) Radar plots showing the overall effects of *R. irregularis* or *T. harzianum* inoculation, with respect to noninoculated plants, on detected GLVs emitted by plants subjected to short‐term herbivory (upper panel) and long‐term herbivory (lower panel). Radar plots display the scaled values for each GLV, where each value is normalized by dividing it by its root mean square. Higher values mean a higher emission (and vice versa). Tables show the scaled area of each polygon relative to the polygon area of the noninoculated treatments. (d, f) Peak intensity of the specific GLVs that showed significant differences according to three‐way ANOVA analyses (after MANOVA analyses, Supporting Information Tables S7, S9): Hexanal, (*Z*)‐3‐hexenyl acetate, (*Z*)‐3‐hexenol, and an unknown GLV with rt of 13.14 min. (e, g) Expression levels of the green leaf volatiles biosynthesis‐related genes *LOXC* (*13‐lipoxygenase C*) and *HPL* (*Hydroperoxide lyase*). GLV emissions and gene expression were analyzed in leaves of plants subjected to short‐term herbivory (1 d, d, e) or long‐term herbivory (10 d, f, g). In (e, g), results are normalized to *SlEF* gene expression levels. In each graph, ‘Herb’ stands for plants with herbivores, and ‘No herb’ stands for plants without herbivores. Plants had been inoculated with *R. irregularis* or *T. harzianum*, or noninoculated. Box plots represent the interquartile range (IQR), the bisecting line represents the median, the whiskers represent 1.5 times the IQR, and the dots represent data points from five individual plants (*n* = 5). Left upper corner of graph panels: Results of two‐way ANOVA. Factors I: inoculation, H: herbivory, and I*H: interaction between inoculation and herbivory. Lines in bold: *P* < 0.05. Following ANOVA analyses, *t*‐test comparisons were also performed between noninoculated and inoculated plants, for either herbivory‐challenged or nonchallenged plants, and significant *P* values are depicted.

These untargeted analyses highlighted GLVs as a prominent chemical group of VOCs driving the separation between herbivory and nonherbivory treatments, both upon short‐ and long‐term herbivory. We thus next performed a targeted VOC analysis approach, focusing specifically on GLVs. The resulting PCA biplots further indicated a clear effect of herbivory on GLV blends, upon both short‐ and long‐term herbivory (Fig. 5a,b). Multivariate analysis of variance (MANOVA) of GLVs (including short‐ and long‐term herbivory datasets) showed that GLV emissions were strongly influenced by herbivory, herbivory time, and the interaction between both factors (Table 1). Microbial inoculation and the interaction between inoculation and herbivory did not affect GLV emissions. Interestingly, there was a significant interaction between microbial inoculation, herbivory, and the time of herbivory (Table 1). These data suggest that the impact of the mutualistic fungi on herbivory‐triggered emission of GLV is affected by how long the plants have been fed upon. We produced radar plots to better visualize the overall effects of microbial inoculation on herbivore‐induced GLVs at the different herbivory times (Fig. 5c). Following short‐term herbivory, polygons corresponding to *R. irregularis*‐colonized and *T. harzianum*‐colonized plants were smaller than to noninoculated plants (Fig. 5c, upper panel). By contrast, after long‐term herbivory, the area of the polygon corresponding specifically to *T. harzianum*‐colonized plants was larger than that of noninoculated plants (Fig. 5c, lower panel).

**Table 1:** Results of multivariate analysis of variance (MANOVA) for the effects of herbivory (H), time (T), inoculation (I), and interactions between them on green leaf volatile emissions.

Variable	Pillai	*f* (df_num_df _den_)	*P* value
Herbivory	0.782	13.985 (10,39)	**< 0.001**
Time	0.807	16.296 (10,39)	**< 0.001**
Inoculation	0.743	1.239 (20,80)	0.246
H × T	0.782	13.958 (10,39)	**< 0.001**
H × I	0.490	1.299 (20,80)	0.205
I × T	0.501	1.339 (20,80)	0.180
H × T × I	0.651	1.929 (20,80)	**0.021**

Values highlighted in bold indicate significant differences at *P* < 0.05.

To assess the impact of the mutualistic microbes on specific GLVs, we conducted three‐way ANOVAs. This analysis revealed a significant or marginally significant (0.05 < *P* < 0.10) interaction between microbial inoculation, herbivory, and herbivory time specifically for the GLVs hexanal (*P* = 0.08, *F* = 2.604) and (*Z*)‐3‐hexenyl acetate (*P* = 0.09, *F* = 2.53) (Table S7). When analyzing microbial inoculants separately, we also found a significant interaction between *R. irregularis* or *T. harzianum* colonization, herbivory, and herbivory time (MANOVA, Table S8). In the case of *Trichoderma*‐colonized plants, three‐way ANOVAs indicated a significant (or marginally significant) interaction between *T. harzianum* colonization, herbivory, and herbivory time for the GLVs hexanal (*P* = 0.064, *F* = 3.69), (*Z*)‐3‐hexenyl acetate (*P* = 0.024, *F* = 5.582), (Z)‐3‐hexenol (*P* = 0.097, *F* = 2.907), and the GLV with rt of 13.14 min (*P* = 0.061, *F* = 3.767) (Table S9). In the case of *R. irregularis*, we did not find significant differences (three‐way ANOVA, *P* < 0.1).

We then focused on the effect of the mutualistic fungi on plant emission of these specific four GLVs (i.e. hexanal; (*Z*)‐3‐hexenyl acetate; (*Z*)‐3‐hexenol; and the GLV with rt of 13.14 min), and on the expression of *LOXC* (encoding a 13‐lipoxygenase C) and *HPL* (encoding a hydroperoxide lyase) genes, involved in the biosynthesis of GLVs. *R. irregularis* colonization, overall, did not affect the emission of the four GLVs nor the expression of the analyzed genes, regardless of the herbivory time (Fig. 5d–g). By contrast, *T. harzianum* colonization affected GLV emission and the expression of *LOXC* and *HPL* differently, depending on the duration of the herbivory (Fig. 5d–g). Upon short‐term herbivory, *T. harzianum* colonization did not affect – or slightly decreased – the emission of the four GLVs (Fig. 5d) nor *LOXC* or *HPL* expression (Fig. 5e). However, upon long‐term herbivory, we found a significantly higher emission of hexanal and (*Z*)‐3‐hexenol (Fig. 5f), and enhanced expression of *LOXC* and *HPL* in *Trichoderma*‐inoculated plants compared with noninoculated plants (Fig. 5g). Notably, herbivory damage level was similar across the different treatments, upon both short‐ and long‐term herbivory (Fig. S2). Altogether, these results indicate that root colonization by the mutualistic fungi, and especially *T. harzianum*, boosts the HPL branch of the 13‐LOX pathway, thereby enhancing the emission of GLVs specifically in plants subjected to long‐term herbivory, while this effect was not observed upon short‐term herbivory. Noticeably, besides GLVs, we also detected four VOCs with rt of 19.84, 20.40, 23.64, and 26.38 min, which were enhanced specifically in microbial‐inoculated plants following long‐term herbivory, when we found an enhanced attraction of parasitoids toward microbial‐inoculated plants (Fig. S4). However, we were unable to identify these specific compounds. We further found that following long‐term herbivory, methyl salicylate was reduced in *Trichoderma*‐inoculated plants compared with that in noninoculated plants (Fig. S4).

## Discussion

Over the last decade, research has revealed that MIR influences plant–herbivore interactions across multiple trophic levels. Indeed, MIR phenotypes have been linked to the induction of both direct and indirect defense traits (Pozo *et al*., 2020). However, these studies have been limited to examining the impact of mutualistic microbes on either direct or indirect defenses in isolation, overlooking potential conflicts and synergisms between MIR‐mediated elicitation of different defense lines. Our findings demonstrate that, in a multitrophic context, MIR expression is associated with priming of both direct and indirect defenses in tomato plants. Notably, MIR phenotypes were adjusted at physiological and molecular levels according to the duration of the herbivory, revealing a new emergent property of MIR that enhances MIR phenotypes.

We found that MIR display resulted in increased mortality of *M. sexta* larvae, which directly reduces herbivore pressure. This enhanced larval mortality was primarily observed in the early larval instars (specifically L1–L3). These specific larval instars correspond to the window of sensitivity of *M. sexta* to induced plant responses, when the larvae are the most sensitive to induced chemical defenses (van Dam *et al*., 2000, 2001). This physiological window occurs before the larvae start to grow exponentially, and consequently, the amount of plant biomass they consume increases (van Dam *et al*., 2001). Induced defenses are proposed to be most beneficial to a plant when they kill or deter the herbivore as quickly as possible. In fact, the faster the herbivore is eliminated, the less damage it can inflict, and the lower the fitness cost for the plant (Moran & Hamilton, 1980; Herms & Mattson, 1992). Our findings indicate that MIR reduces *M. sexta* damage as it enhances mortality during the early larval stages.

To better understand the mechanistic basis underlying MIR impact on direct defenses, we focused on the AOS branch of the 13‐LOX pathway, which is the core regulator of direct antiherbivore defenses through the biosynthesis of jasmonates (Erb & Reymond, 2019; Farmer & Goossens, 2019). As expected, we found that plants activated the AOS branch upon attack regardless of microbial inoculation. This was evidenced by the induced accumulation of jasmonates and jasmonate‐regulated defense genes in leaves following short‐ and long‐term herbivory. Defense responses were generally lower after long‐term herbivory, likely reflecting a transient response or less intense feeding over time. Importantly, the mutualistic fungi, and more strongly *T. harzianum*, boosted this herbivore‐induced jasmonate‐related response. We currently lack a clear explanation for the differing effects observed between the two mutualistic fungi. However, it has been reported that microbial genotype plays a key role in modulating MIR phenotypes (Fiorilli *et al*., 2024). Strikingly, the MIR‐boosted response was limited to early herbivory and not observed in plants subjected to long‐term herbivory. Collectively, our findings demonstrate that MIR display is related to priming of the AOS branch of the 13‐LOX pathway, resulting in a boosted activation of jasmonate‐regulated defenses upon herbivore attack. In line with the larval survival data, this priming effect is specific to the early stages of herbivory and aligns with the period when *M. sexta* are most sensitive to induced defenses and before larvae enter the exponential growth phase. While we focused on jasmonates and key defense‐related genes, future research integrating comprehensive metabolomics analyses and controlled mechanical damage experiments may provide a deeper understanding of MIR‐induced shifts in leaf chemistry, and the underlying molecular mechanisms.

As a general rule, plants deploy multiple defense strategies to fend off herbivores, involving both direct and indirect defenses (Edwards *et al*., 2023). In the case of *M. sexta*, natural enemies such as parasitic wasps and predators heavily impact their survival rates (Garvey *et al*., 2020). One of the most common natural enemies of *M. sexta* is the parasitic wasp *C. congregata*, which exerts a strong negative survival pressure, killing up to 50% of *M. sexta* larvae (Jacobsen, 2021). As expected, *M. sexta* herbivory elicited indirect defenses, promoting the attraction of *C. congregata* upon short‐term and long‐term herbivory, regardless of microbial inoculation. Notably, we found that root colonization by the mutualistic fungi further enhanced parasitoid attraction, but only for plants that were subjected to long‐term herbivory. In our bioassay experiment, at this specific period of herbivory (10 d), *M. sexta* larvae had reached the third instar, which aligns with the instars preferred for *C. congregata* oviposition (Kingsolver *et al*., 2012). Although *M. sexta* larvae can exhibit increased mobility at these later stages (Kessler & Baldwin, 2002), in our Y‐tube bioassays, they remained on the feeding plant, preventing us from assessing whether larval movement further influences MIR effects on parasitoid recruitment. Overall, our results indicate that the beneficial fungi improve *C. congregata* parasitism efficiency. However, while microbe‐induced priming of direct defenses was limited to short‐term herbivory, microbe‐induced indirect defenses were evident specifically upon long‐term herbivory.

The release of herbivore‐induced VOCs by plants drives the attraction of natural enemies of herbivores (Aartsma *et al*., 2017). *M. sexta* herbivory strongly altered the blends of VOCs, in particular the emission of green leaf volatiles (GLVs), indole, and terpenes, which are involved in the attraction of parasitoids and predators (Aartsma *et al*., 2017). It is remarkable that the impact of the mutualistic microbes on herbivore‐induced VOCs was relatively mild. However, *C. congregata*, as well as other parasitic wasps, has evolved the ability to perceive small differences in the blends of VOCs associated with high‐quality hosts (Danner *et al*., 2018). Our study demonstrates that the mutualistic microbes, and more strongly *T. harzianum*, led to a modulation of herbivore‐induced GLV emissions. Specifically, upon long‐term herbivory, *T. harzianum* inoculation boosted the emission of hexanal and (*Z*)‐3‐hexenol and increased expression of the genes *HPL* and *LOXC*, involved in the production of GLVs via the HPL branch of the 13‐LOX pathway (Shen *et al*., 2014; Matsui & Engelberth, 2022). It has been proposed that GLVs are reliable indicators of herbivory, as their presence indicates active feeding (Joo *et al*., 2018). Indeed, certain GLVs such as hexanal, hexenol, and hexenyl acetate enhance the attraction of the *C. congregata* relatives *C. maginiventris*, *C. glomerata*, and *C. kariyai* (Mandour *et al*., 2011; Ngumbi & Fadamiro, 2012; Blažytė‐Čereškienė *et al*., 2022). Although we cannot exclude the possibility that other VOCs, or specific volatile blends, may be involved in the observed MIR phenotype, we postulate that the boosted emission of GLVs in plants subjected to long‐term herbivory is likely involved in the enhanced attraction of *C. congregata* wasps, mediated by *T. harzianum* root colonization. Functional analysis using genetic approaches, as described in other systems (López‐Gresa *et al*., 2018), might help to further dissect the contribution of specific GLVs to MIR phenotypes. Additionally, microbes can induce changes in the plant nutritional status, and this can potentially have an impact on herbivore performance or on parasitoid attraction (Pozo *et al*., 2020). Even though several MIR‐related studies have disentangled nutritional modulation from resistance induction in tomato and other plants (Martínez‐Medina *et al*., 2013; Pozo de la hoz *et al*., 2021; Dejana *et al*., 2022), we cannot rule out that some alterations in nutritional aspects may have contributed to the observed MIR phenotypes. This possibility warrants further investigation in future studies.

Taken together, our findings indicate that in a multitrophic context including *M. sexta* and *C. congregata*, MIR expression involves the elicitation of direct defenses through priming the AOS branch of the 13‐LOX pathway, and indirect defenses through priming the HPL branch. The deployment of these two defense strategies is plastically adjusted at the molecular and physiological levels to the duration of herbivory. It has been reported that the HPL and AOS branches of the 13‐LOX pathway crosstalk to fine‐tune plant responses to a diverse range of perturbations (Matsui & Engelberth, 2022). Moreover, several studies have suggested that substrate competition exists between the AOS and HPL pathways (Halitschke *et al*., 2004; Wang *et al*., 2020). Based on this, it is tempting to speculate that the distinct temporal priming of the HPL and AOS branches by MIR may promote complementarity, rather than conflicts, between MIR‐driven elicitation of direct and indirect defense strategies.

Still, the eventual impact of MIR display on the performance of natural enemies was hard to predict, as it can lead to an enhanced or reduced quality of herbivorous hosts to their natural enemies (Martínez‐Medina *et al*., 2024). Indeed, our study shows that the offspring of *C. congregata* performed better when developing in *M*. *sexta* hosts fed on MIR‐displaying plants, even though their hosts performed worse on these plants. Previous research demonstrated that feeding on MIR‐expressing plants results in a rearrangement of the herbivore's metabolic and transcriptomic profiles, leaving an MIR imprint in surviving larvae (Papantoniou *et al*., 2021; Di Lelio *et al*., 2023; Martínez‐Medina *et al*., 2024). Specifically, *M. sexta* larvae fed on MIR‐expressing tomato plants contain higher levels of toxic plant compounds, such as glycoalkaloids, in their gut and fat bodies (Papantoniou *et al*., 2021). Intriguingly, these metabolic changes did not negatively affect *C. congregata* attraction, or parasitoid performance. These results may be related to the specialization degree of *C. congregata*, which is specialized on *M. sexta*, a species that commonly feeds on alkaloid‐containing plants (Harvey *et al*., 2007). By contrast, higher levels of direct defenses may have contributed to the suppression of immune responses in the herbivorous hosts, as shown in other systems, thereby increasing the chances of successful parasitization (Harvey, 2005; Ghosh *et al*., 2023). Our results suggest potential defensive synergisms between MIR‐triggered direct and indirect defenses, as MIR enhanced the attraction of the parasitoids, and the effect of MIR expression in *M. sexta* larvae was positively linked to parasitoid performance. This synergistic expression of both MIR elicitation of direct and indirect traits may eventually lead to a more effective control of *M. sexta* larvae and an enhanced MIR phenotype.

**Fig. 6:**
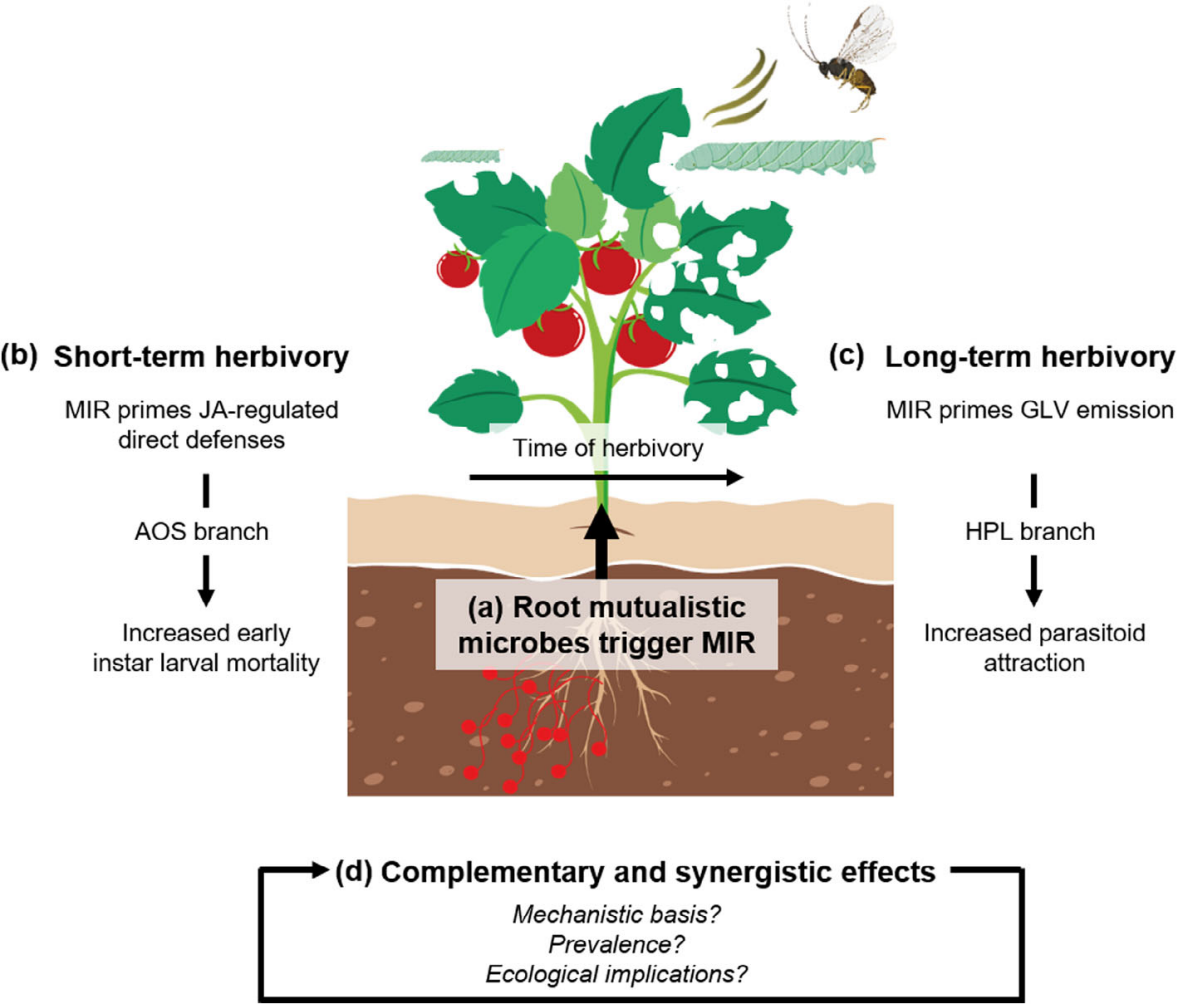
MIR involves priming of direct or indirect defenses depending on the timing of herbivory (a) Root‐associated mutualistic microbes (in red) trigger MIR. (b) Under short‐term herbivory, MIR phenotypes are linked to enhanced JA‐regulated direct defenses via priming of the allene oxide synthase (AOS) branch of the oxylipin pathway, increasing early‐instar larval mortality. (c) Under long‐term herbivory, MIR phenotypes are associated with increased emission of green leaf volatiles (GLVs) via priming of the hydroperoxide lyase (HPL) branch, enhancing parasitoid attraction. (d) This time‐dependent modulation may promote complementary and synergistic effects between direct and indirect defenses. As shown in the figure (in italics), there are still unresolved questions regarding whether this ‘MIR‐switching’ represents an active, microbe‐mediated process or a passive amplification of existing plant responses; how widespread this phenomenon is across systems, and its ecological implications. The specific elements *Manduca sexta* and *Cotesia congregata* of this figure were created in BioRender (https://app.biorender.com/illustrations/6338c751a2f20ef653f5bc99).

In conclusion, our study indicates that within multitrophic contexts, MIR expression involves a complex array of defense‐related traits, comprising both direct and indirect defense strategies that dynamically adjust to the temporal pattern of herbivory (Fig. 6). Although the mechanistic basis of this dynamic response remains unresolved, our results indicate that coordination of the different defense strategies may foster complementary and synergistic interactions between MIR‐elicited direct and indirect defenses, thereby enhancing MIR phenotypes. Further research into the functioning and ecological implications of this dynamic response should reveal the generality of this phenomenon beyond our specific biological model. In particular, we cannot predict how it would affect tri‐trophic interactions in systems where parasitoids or predators targeting early‐instar larvae prevail, or under herbivory by late instar caterpillars that are less sensitive to direct defenses. Under these scenarios, potential trade‐offs might emerge, compromising the effectiveness of MIR. While the prevalence and the ecological implications of this response remain to be determined (Fig. 6), we propose that it represents a novel emergent property that becomes apparent only when MIR phenotypes are understood through the lens of the intricate multitrophic interactions under which plant–microbe associations have evolved.

## Competing interests

None declared.

## Author contributions

JR and IF contributed equally. NMD and AMM contributed equally. MJP, NMD and AMM planned and designed the research. JR, IF, AJT, AW and AMM performed the experiments. JR, IF, FJC, AW, PMRB, KK, MJP and AMM analyzed the data. AMM and NMD secured the funding and wrote the manuscript with the input from all the authors.

## Disclaimer

The New Phytologist Foundation remains neutral with regard to jurisdictional claims in maps and in any institutional affiliations.

## Supporting information


**Fig. S1** The impact of root colonization by *Rhizophagus irregularis* or *Trichoderma harzianum* on shoot and root biomass, and number of flowers.
**Fig. S2** The impact of root colonization by *Rhizophagus irregularis* or *Trichoderma harzianum* on *Manduca sexta* eaten leaf area and plant biomass.
**Fig. S3** The impact of root colonization by *Rhizophagus irregularis* or *Trichoderma harzianum* on the volatile blends emitted by tomato plants in response to *Manduca sexta* short‐ and long‐term herbivory.
**Fig. S4** The impact of root colonization by *Rhizophagus irregularis* or *Trichoderma harzianum* on selected volatiles.
**Methods S1** Real‐time quantitative reverse transcription PCR.
**Methods S2** Determination of jasmonate concentrations.
**Methods S3** Volatile collection, gas chromatography, and volatile data processing.
**Methods S4** Assessment of the impact of root colonization by the fungal mutualists on parasitoid behavior.
**Methods S5** Assessment of the impact of root colonization by the fungal mutualists on parasitoid performance.
**Methods S6** Statistical analysis.

**Table S1** Specific primers used for cDNA pre‐amplification and relative expression quantification by qRT‐PCR.
**Table S2** Results of three‐way ANOVA for the effects of herbivory, time, inoculation, and interactions between them on the production of jasmonate‐regulated defenses.
**Table S3** Peak area from the volatile organic compounds detected in tomato leaves during short‐term herbivory.
**Table S4** Peak area from the volatile organic compounds detected in tomato leaves during long‐term herbivory.
**Table S5** Compounds with significant differences among treatments upon short‐term (1 d) *Manduca sexta* herbivory.
**Table S6** Compounds with significant differences among treatments upon long‐term (10 d) *Manduca sexta* herbivory.
**Table S7** Results of three‐way ANOVA for the effects of herbivory, time, inoculation, and interactions between them on the emission of the green leaf volatiles hexanal and (Z)‐3‐hexenyl acetate.
**Table S8** Results of multivariate analysis of variance for the effects of herbivory, time, inoculation, and interactions between them on green leaf volatile emissions.
**Table S9** Results of three‐way ANOVA for the effects of herbivory, time, inoculation (with *Trichoderma harzianum*), and interactions between them on the emission of the green leaf volatiles hexanal and (Z)‐3‐hexenyl acetate.
Please note: Wiley is not responsible for the content or functionality of any Supporting Information supplied by the authors. Any queries (other than missing material) should be directed to the *New Phytologist* Central Office.

## Data Availability

All data are available within the article and Tables S3 and S4. Sequence data used in this article can be found in the Solgenomics platform (https://solgenomics.net/) under the following ITAG4 accession nos.: Solyc09g089500; Solyc01g095200; Solyc11g021060; Solyc03g020060; Solyc00g071180; Solyc12g010020; Solyc01g006540; Solyc07g049690; Solyc06g009960; Solyc03g078400; Solyc04g081490. Raw data of volatile datasets are available in Zenodo repository (https://zenodo.org/records/8139524) under doi: 10.5281/zenodo.8139523.
