# Supplementary material for: Microbe‐induced resistance involves priming of direct or indirect defenses according to the stage of herbivory
Source: New Phytol. 2025 Oct 29;248(6):3256–69. doi: 10.1111/nph.70652 (PMC12630452; doi:10.1111/nph.70652)
Supplement: Supplementary file 1 — Fig. S1 The impact of root colonization by Rhizophagus irregularis or Trichoderma harzianum on shoot and root biomass, and number of flowers. Fig. S2 The impact of root colonization by Rhizophagus irregularis or Trichoderma harzianum on Manduca sexta eaten leaf area and plant biomass. Fig. S3 The impact of root colonization by Rhizophagus irregularis or Trichoderma harzianum on the volatile blends emitted by tomato plants in response to Manduca sexta short‐ and long‐term herbivory. Fig. S4 The impact of root colonization by Rhizophagus irregularis or Trichoderma harzianum on selected volatiles. Methods S1 Real‐time quantitative reverse transcription PCR. Methods S2 Determination of jasmonate concentrations. Methods S3 Volatile collection, gas chromatography, and volatile data processing. Methods S4 Assessment of the impact of root colonization by the fungal mutualists on parasitoid behavior. Methods S5 Assessment of the impact of root colonization by the fungal mutualists on parasitoid performance. Methods S6 Statistical analysis. [file NPH-248-3256-s001.pdf]

***New Phytologist* Supporting Information**

Article title: **Microbe-induced resistance involves priming of direct or indirect defenses according to the stage of herbivory**

Authors: Javier Rivero; Iván Fernández; Francisco J. Colina; Axel J. Touw; Alexander Weinhold; Pablo M. Rodríguez-Blanco; Karen Kester; María J. Pozo; Nicole M. van Dam, Ainhoa Martínez-Medina

Article acceptance date: 11 September 2025

The following Supporting Information is available for this article:

Figure S1. The impact of root colonization by *Rhizophagus irregularis* or *Trichoderma harzianum* on shoot and root biomass, and number of flowers.
Figure S2: The impact of root colonization by *Rhizophagus irregularis* or *Trichoderma harzianum* on *Manduca sexta* eaten leaf area and biomass
Figure S3. The impact of root colonization by *Rhizophagus irregularis* or *Trichoderma harzianum* on the volatile blends emitted by tomato plants in response to *Manduca sexta* short- and long-term herbivory.
Figure S4. The impact of root colonization by *Rhizophagus irregularis* or *Trichoderma harzianum* on selected volatiles.
Methods S1: Real-time quantitative reverse transcription PCR.
Methods S2: Determination of jasmonate concentrations.
Methods S3: Volatile collection, gas chromatography, and volatile data processing.
Methods S4: Assessment of the impact of root colonization by the fungal mutualists on parasitoid behavior.
Methods S5: Assessment of the impact of root colonization by the fungal mutualists on parasitoid performance.
Methods S6: Statistical analysis.
Supporting Information References.

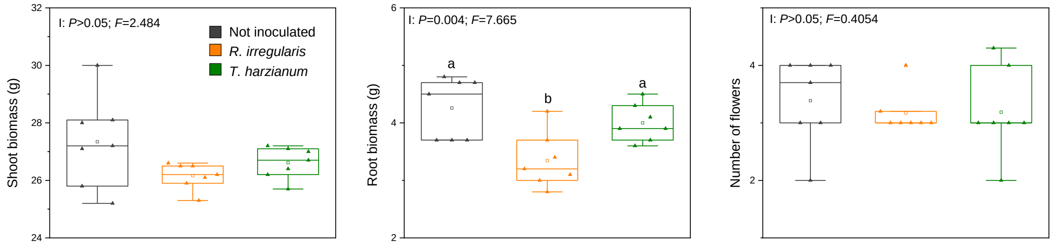

Fig. S1 The impact of root colonization by *Rhizophagus irregularis* or *Trichoderma harzianum* on shoot and root biomass, and number of flowers. The fresh biomass of shoots (left panel) and roots (middle panel), and the number of flowers (right panel) were assessed in tomato plants that had been inoculated with *Rhizophagus irregularis* or *Trichoderma harzianum* or not inoculated. Box plots represent the IQR, the bisecting line represents the median, the whiskers represent 1.5 times the IQR, and the dots represent data points from seven individual plants. I: factor inoculation. In the middle panel, different letters indicate differences between treatments (ANOVA, Tukey's test; *P* <0.05).

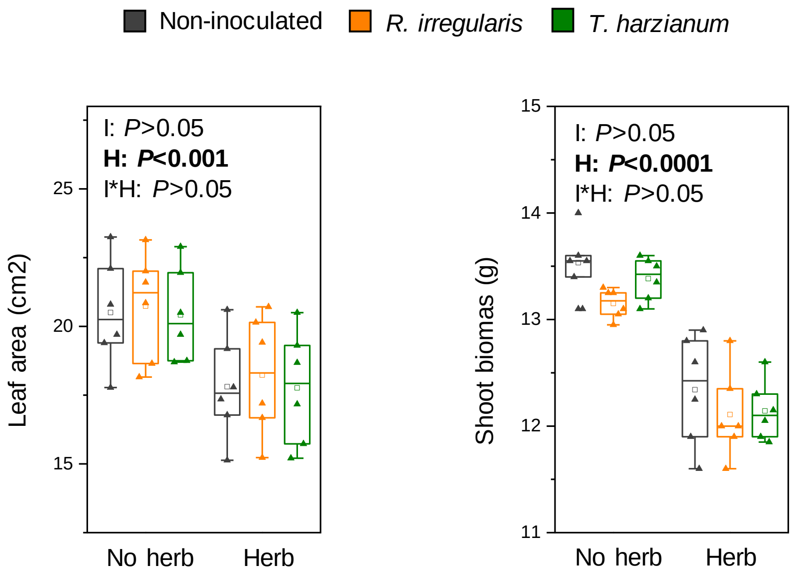

Fig. S2 The impact of root colonization by *Rhizophagus irregularis* or *Trichoderma harzianum* on *Manduca sexta* eaten leaf area and plant biomass. Leaf area (left panel) was measured in leaflets of tomato plants not challenged with the herbivores (No herb) or after 24 hours of *M. sexta* herbivory (Herb). Leaflets were photographed and leaf area consumed was calculated using the ImageJ software (https://imagej.nih.gov/). Shoot biomass (right panel) was assessed in plants not challenged with the herbivores (No herb) or after 10 days of *M. sexta* herbivory (Herb). Plants had been inoculated with *R. irregularis* or *T. harzianum*, or non-inoculated. Box plots represent the interquartile range (IQR), the bisecting line represents the median, the whiskers represent 1.5 times the IQR, and the dots represent data points from six individual plants (n=6). Left upper corner of graph panels: results of two-way ANOVA. Factors I: inoculation, H: herbivory, and I*H: interaction between inoculation and herbivory. Lines in bold: *P* value <0.05.

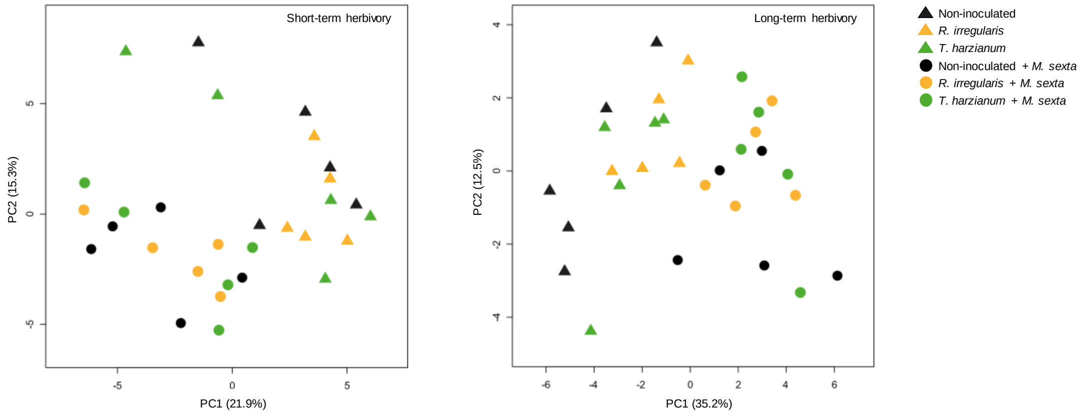

Fig. S3 The impact of root colonization by *Rhizophagus irregularis* or *Trichoderma harzianum* on the volatile blends emitted by tomato plants in response to *Manduca sexta* short- and long-term herbivory. Two-dimensional principal component analysis (PCA, n=5) of volatile blends emitted by leaves of plants without herbivores (triangles), and by plants subjected to herbivory by *M. sexta* (circles). Volatiles blends were analyzed upon short-term (1 day, left panel), or long-term (10 days, right panel) following herbivory.

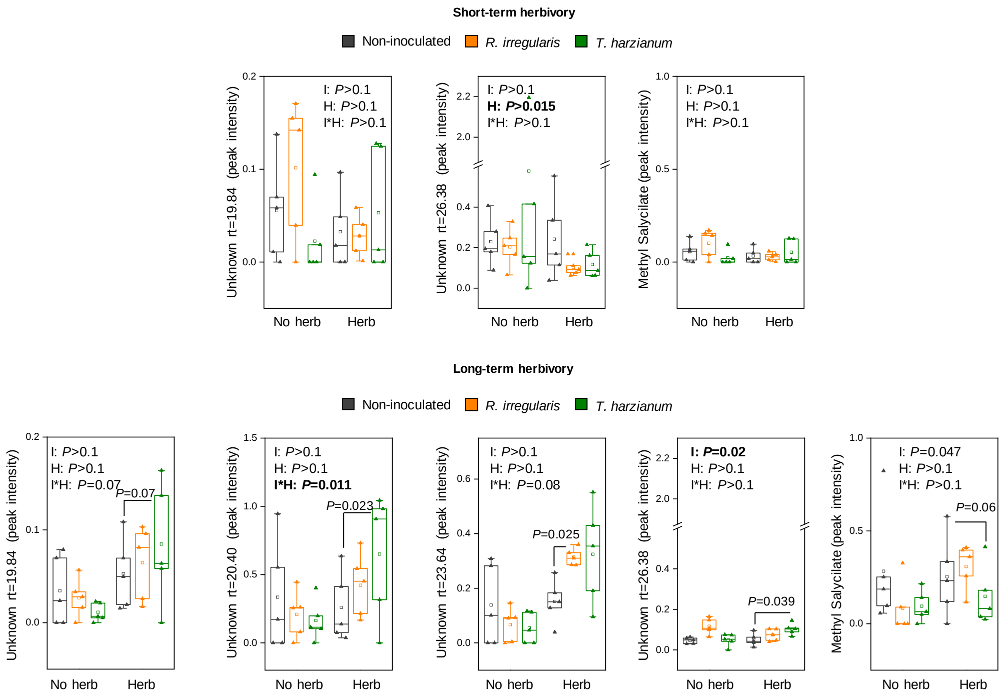

Fig. S4 The impact of root colonization by *Rhizophagus irregularis* or *Trichoderma harzianum* on selected volatiles. Peak intensity of selected unknown volatiles with retention time (rt) of 19.84 min, 26.38 min, and methyl salicylate emitted by plants upon short-term herbivory (1 day, upper panel); and unknown volatiles with rt of 19.84 min, 20.40 min, 23.64 min, 26.38 min, and methyl salicylate emitted by plants upon long-term herbivory (10 days, lower panel). In each graph, Herb stands for plants with herbivores, and No herb stands for plants without herbivores. Plants had been inoculated with *R. irregularis* or *T. harzianum*, or non-inoculated. Box plots represent the interquartile range (IQR), the bisecting line represents the median, the whiskers represent 1.5 times the IQR, and the dots represent data points from five individual plants. Left upper corner of graph panels: Results of two-way ANOVA. Factors I: inoculation, H: herbivory, and I*H: interaction between inoculation and herbivory. Lines in bold: *P* value <0.05. Following ANOVA analyses, *t*-test comparisons were performed between non-inoculated and inoculated plants, for herbivory-challenged plants, and significant *P* values are depicted.

Methods S1 Real-time quantitative reverse transcription PCR. Total RNA from fresh leaves was extracted by using the RNeasy Plant Mini Kit (Qiagen) and treated with DNase I (Qiagen) according to the manufacturer's instructions. Five random independent replicates were analyzed per treatment. First-strand cDNA was synthesized from 0.5 µg using PrimeScript RT reagent kit (Takara). Quantitative PCR was conducted using the SYBR PREMIX EX TAQ (Takara) following the manufacturer's instructions and a thermal cycler (Bio-RAD, CFX Opus 384) with the following cycling program: 30 sec 95°C, 40 cycles of 5 sec 95°C and 60°C 34 seq, followed by a dissociation process of 95°C 15 seq, 60°C 1 min and 95°C 15 seq. The sequences of the gene-specific primers are shown in Table S1. Before normalization, we analyzed the expression of three different tomato housekeeping genes under the different conditions of our study: actin (Solyc03g078400), β-tubulin (Solyc04g081490), and elongation factor 1-α (Solyc06g009960). We used the Normfinder software (https://moma.dk/normfinder-software) to find the optimal normalization gene. According to the results, expression values were normalized using the housekeeping gene elongation factor 1-α (*EF-1α*). The normalized data were further processed by the $2^{-\Delta\Delta ct}$ method (Livak & Schmittgen, 2001).

Methods S2 Determination of jasmonate concentrations. We extracted plant hormones from fresh leaves using ethyl acetate containing the internal standards: 40 ng D_6-JA and 40 ng D_6-JA-Ile as the solvent, according to Escobar-Bravo *et al*. (2018). The levels of 12-*oxo*-phytodienoic acid (OPDA), jasmonic acid (JA) and jasmonyl-*L*-isoleucine (JA-Ile) were analyzed by using liquid chromatography (Bruker Advance UHPLC, Bremen, Germany) coupled to a mass spectrometer (Bruker Elite EvoQ Triple quadrupole, Bremen, Germany) (LC/MS EVOQ) as described in Escobar-Bravo *et al*. (2019). The separation was achieved on a Zorbax Eclipse XDB-C18 column (4.6 x 50 mm, 1.8 µm, 80 Å, Agilent technologies, Santa Clara, CA, USA). Data acquisition and processing were performed using the 'MS Data Review' software (Bruker MS Workstation, version 8.2). The same five replicates as for transcriptomic analyses were analyzed per treatment. Phytohormone levels were calculated based on the peak area of the corresponding internal standard and the amount of fresh weight of the leaf material (ng^{-1} mg^{-1} FW).

Methods S3 Volatile collection, gas chromatography, and volatile data processing. The corresponding apical leaflets (according to the experimental design described in the main document) were enclosed in two transparent 50-ml plastic containers with hair clips as illustrated in the figure below. The insects remained feeding on the corresponding treatments during volatile collection. In addition, we also sampled volatiles from empty plastic containers. Those 'air blanks' were used in further data processing to exclude systemic contaminations. Stainless steel thermal desorption sorbent tubes, loaded with 200 mg of Tenax (MARKES, Llantrisant, United Kingdom) were inserted into one of the plastic cups and connected to a vacuum pump system. A charcoal filter was included to avoid contamination. Charcoal-filtered air was pulled for 20 min through the thermal desorption sorbent tubes at a flow rate of 0.5 L min^{-1}, resulting in a total of 10 L of sampled headspace air per biological replicate. Airflow was created using a diaphragm vacuum pump (KNF, model Laboport n820.3ft.18). Airflow was controlled using a setup of 12 analog airflow controllers (Brooks instruments, model 2510). For the entire bioassay, we collected volatiles from the leaves of 72 plants, 36 for short-term herbivory and 36 plants for long-term herbivory bioassays. Volatile blends were analyzed by a thermal desorption gas chromatograph-mass spectrometer (TD-GC-MS) consisting of a thermos desorption unit (MARKES, Unity 2, Llantrisant, United Kingdom) equipped with an autosampler (MARKES, Ultra 50/50), as described in Fernández *et al*. (2024). Tubes were desorbed with helium as carrier gas and a flow path temperature of 150°C using the following conditions: Dry Purge 5 min at 20

ml/min, Pre Purge 2 min at 20 ml/min, Desorption 8 min at 280°C with 20 ml/min, Pre Trap fire purge 1 min at 30 ml/min, Trap heated to 300°C and hold for 4 min. Volatiles were separated on a gas chromatograph (Bruker, GC-456, Bremen, Germany) connected to a triple-quad mass spectrometer (Bruker, SCION). Separation took place on a DB-5MS column (30 m x 0.25 mm x 0.25 µm. Restek, Germany). The conditions of the GC were as follows: 40°C for 5 min, 5C/min to 185°C, 30C/min to 260, and hold for 0.5 min. The mass spectrometer was operated in full scan mode with the following parameters: transfer line temperature 280°C, ion source temperature 260°C, scan time 250 ms, scan range 40-550 m/z, ionization 70 eV. We only considered peaks with a signal-to-noise ratio > 10. Peaks that were also present in air blanks were regarded as systemic contamination and were excluded from further analysis. The peak areas of the remaining m/z signals were calculated using the Bruker Workstation software (v8.0.1). Tentative identification was carried out using the NIST 2005 mass spectral library. Comparisons between calculated Kovats retention indices and those published in the literature and databases were also used for tentative identification. The following specific standards were also included: α-Pinene; β-Pinene; Ethyl benzoate; Eucalyptol; Linalool; Methyl salicylate; Myrecene; Limonene; and Trans caryophyllene. Relative quantification (peak areas of individual compounds) was obtained using a single (target) ion. The individual peak areas of each compound were further used in the statistical analysis, by using the Metaboanalyst web server for metabolomics data analysis (Xia & Wishart, 2016). For the statistical analyses, the same five replicates as for transcriptomic and metabolomic analyses were used.

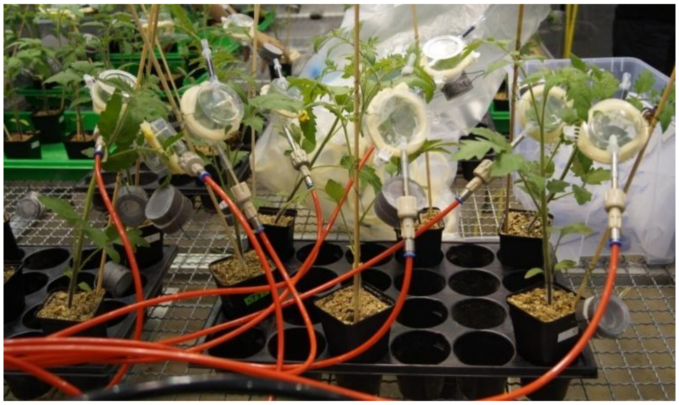

Set-up used for volatile collection

Methods S4 Assessment of the impact of root colonization by the fungal mutualists on parasitoid behavior. Air was pumped into a flask containing activated carbon to first purify the air and then passed through the jars containing single plants, at the end of each arm of the Y-tube at a flow rate of 0.2 LPM (Ayers, 2015). The set-up was placed on a table surrounded by white cardboard to reduce any visual bias for the parasitic wasps. All the experiments were conducted in an acclimatized room (26±1°C) between 10 a.m. and 3 p.m., intervals chosen to coincide with periods of peak activity (Ayers, 2015). Before each experiment, parasitoids and plants were placed in the acclimatized room for at least 1 hour to allow acclimatization. The plants were introduced in the jars, and a single female *C. congregata* wasp was released in the Y-tube. *Manduca sexta* larvae remained feeding on the plants during the experiments. Wasps were given 6 min to make a choice. A choice was recorded when a female wasp reached a line marked 1 cm from the end of each olfactometer arm and did not return to the junction for at least 10 uninterrupted seconds, according to Ayers (2015). Wasps that did not choose within 6 min, or that spent

less than 10 uninterrupted seconds on either olfactometer arm were counted as non-responders and excluded from statistical analyses. After 5 wasps were tested, the position of the jars containing the plants was interchanged to compensate for any unforeseen asymmetry in the set-up. After 10 wasps were tested the Y-tube was cleaned and rotated. Experiments were repeated on several days, with ca. 15 female wasps tested per pairwise comparison per day. In total, 4-6 sets of plants and 50–100 females were evaluated per pairwise comparison. Due to the logistical limitations and to make the best use of our biological resources, we chose to stop further testing of specific treatments after about 50 mated females had been tested and no significant differences were observed. Given the complexity of the system involving three trophic levels and microbial treatments, and to further ensure the robustness of our conclusions, we continued testing in conditions where significant effects were emerging. This allowed us to confirm the consistency and reliability of the observed patterns.

Methods S5 Assessment of the impact of root colonization by the fungal mutualists on parasitoid performance. Naïve, mated female *C. congregata* wasps (2-4 days old) were used to parasitize third-instar *M. sexta* larvae. To avoid possible effects of root colonization by *R. irregularis* or *T. harzianum* on parasitoid load, *M. sexta* larvae were reared in an artificial diet for 7 days, until reaching instar L3. Parasitism was achieved by exposing *M. sexta* larvae individually to adult wasps at room temperature (26±1°C) in the wasp colony enclosure, and removing the larvae once parasitization was observed (oviposition event lasting more than 3 seconds, as described by Moore *et al*., 2020). Individual parasitized *M. sexta* larvae were then randomly placed on not inoculated plants, or on plants inoculated with *R. irregularis* or *T. harzianum*. The parasitized *M. sexta* larvae were placed in the third fully expanded leave (counted from the top) with one larva per plant, and allowed to feed *ad libitum* on the entire plant. Individual plants were covered with mesh bags. Plants were kept in a climate room at 25 ± 1°C, with a 16-h light : 8-h dark cycle, and 70% relative humidity. During the bioassay, plants were exchanged as needed before all of the leaf material had been consumed. Parasitized caterpillars were observed daily until cocoons were formed. We recorded the time until wasp emergence (i.e., second-instar wasp larvae emerging from *M. sexta* cuticle). The percentage of survival of parasitized *M. sexta* larvae was also recorded. Once emerged, cocoons from each parasitized *M. sexta* larvae were counted and weighed. Cocoons were kept in glass vials closed with cotton wool until adult parasitoids emerged at 22 ± 2 °C, with a 12-h light : 12-h dark cycle, and 50% relative humidity. Emerged wasps were counted and the percentage of hatching wasps from the total number of cocoons was calculated.

Methods S6 Statistical analysis. Datasets were analyzed by using R software v 3.6.1 (R Core Team, 2021). One-way ANOVA, two-way ANOVA and three-way ANOVA linear models were performed to analyze datasets of gene expression, phytohormonal content, parasitoid performance and volatiles. Normality of data distribution and homogeneity of variance were verified using Shapiro–Wilk and Levene's tests, respectively. When data did not meet these assumptions, square-root and log transformations were applied. Tukey's test was used for overall comparisons among treatment groups, and *t*-tests were used for pairwise comparisons. We used an overall threshold of $P < 0.05$, except for volatiles data that we used $P < 0.1$. We decided this approach to capture potentially biologically relevant trends that might otherwise be missed with a stricter cutoff. *Manduca sexta* survival was compared among treatments using a Kaplan-Meier survival analysis, and significant differences were assessed by using log-rank tests and Cox regression models. A binomial test (p=q=0.5, two-tailed, α=0.05) was used to determine *C. congregata* preference in the choice assays. Statistical analysis of volatiles was performed using the Metaboanalyst web server v 4.0 (Xia &

Wishart, 2016). For each feature, the average peak area of the blanks was subtracted from the corresponding peak area. After the filtering step, samples were log normalized and pareto scaled. Principal component analysis (PCA) was performed over processed peak intensity MS data. Multivariate analysis of variance (MANOVA) was performed over green leaf volatile datasets. The full model included herbivory, time, inoculation, and interactions between them. Following MANOVA, three-way ANOVA linear models were performed for each green leaf volatile. Radar plots were produced using the R function *ggradar* in ggplot2 using scaled values, whereby each value was normalized by dividing it by its root mean square. The area of each polygon generated was calculated using Fiji.

Supporting Information References

Ayers M. 2015. Behavioral responses of male parasitic wasps to plant cues: a comparison of two host-plant complex sources of *Cotesia congregata* (Say). *PhD Thesis, Virginia Commonwealth University, Richmond, VA, USA.* doi.org/10.25772/D6SM-K525.

Escobar-Bravo R, Chen G, Kim HK, Grosser K, van Dam NM, Leiss KA, Klinkhamer PGL. 2018. Ultraviolet radiation exposure time and intensity modulate tomato resistance to herbivory through activation of jasmonic acid signaling. *Journal of Experimental Botany* **70**: 315-327.

Fernández I, Bouffaud ML, Martínez-Medina A, Schädler M, Tarkka MT, Weinhold A, van Dam NM, Herrmann S, Buscot F. 2024. Endogenous rhythmic growth and ectomycorrhizal fungi modulate priming of antiherbivore defences in subsequently formed new leaves of oak trees. *Journal of Ecology* **00**: 1-15.

Livak KJ, Schmittgen TD. 2001. Analysis of relative gene expression data using real-time quantitative PCR and the 2(-Delta Delta C(T)) Method. *Methods* **25**: 402-408.

Moore ME, Kester KM, Kingsolver JG. 2020. Rearing temperature and parasitoid load determine host and parasitoid performance in *Manduca sexta* and *Cotesia congregate*. *Ecological Entomology* **45**: 79-89.

Xia J, Wishart DS. 2016. Using MetaboAnalyst 3.0 for comprehensive metabolomics data analysis. *Current Protocols in Bioinformatics* **55**: 14.10.1- 14.10.91.
